# Classification of intraarticular calcaneal fractures: comparison of two classification systems

**DOI:** 10.1007/s00590-023-03800-x

**Published:** 2024-01-24

**Authors:** Mirosław Falis, Andrzej Bargiel, Krystian Pyszel, Patrick Simon

**Affiliations:** 1 Department of Traumatology and Orthopedic Surgery, Municipal Hospital in Ostrów Wielkopolski, Limanowskiego 20/22 St, 63-400 Ostrów Wielkopolski, Poland; 2 PhD Honorary Professor Orthopedic and Trauma Surgery, 2 14 Rue Victor Hugo, 69002 Lyon, France

**Keywords:** 3D CT imaging, Calcaneal fractures, Reliability, Intraobserver agreement, Interobserver reliability

## Abstract

**Purpose:**

Accurately classifying displaced intraarticular calcaneal fractures (DIACFs) is essential for orthopedic surgeons to choose optimal treatment methods and provide results evaluation and communication. Many authors studying used Sanders classification reported moderate intra- and interobserver reliability. Taking the software opportunity of 3D virtual exarticulation, Goldzak updated French tri-dimensional Utheza classification, providing an alternative framework for classifying DIACFs. The aim of this study was to compare the intra- and interobserver reliability of Sanders versus Goldzak classification systems.

**Methods:**

The CT scans of 30 patients with displaced intraarticular calcaneal fractures, treated in the same trauma center between 2014–2018, were analyzed by 16 medical doctors (specialists and residents in orthopedic surgery, specialists and residents in radiology), and classified according to Sanders and Goldzak classifications. The same images were sent on two separate sessions, in a randomized order. Interobserver reliability and intraobserver reproducibility were assessed using Kappa statistics and Gwet’s AC1 coefficient.

**Results:**

Interobserver reliability using Gwet reported a value of 0.36 for Goldzak classification and 0.30 for Sanders classification (corresponding to “fair assessment” in both cases). In absence of subclasses, “substantial assessment” was reported for Goldzak classification (Gwet of 0.61) and “moderate assessment” for Sanders classification (Gwet of 0.46). Goldzak system had a greater interobserver reliability in the group of radiology residents. Intraobserver reliability coefficient was 0.60 for Goldzak classification and 0.69 for Sanders classification, indicating a substantial agreement for both classifications.

**Conclusion:**

Despite the better view of the fracture lines provided by 3D reconstructions, this study failed to prove the superiority of Goldzak classification compared to Sanders classification for DIACFs.

## Introduction

Fracture classifications are proposed to clarify fracture patterns and highlight treatment guidelines. CT scans have been in use since the 1990s to aid the analysis of complex fracture pathology. Although Sanders classification is the most used system for classifying displaced intraarticular calcaneal fractures (DIACFs) over the world, the assessment of this classification was fair in all published studies [1–6] and its usefulness (especially the subtypes: A, B, and C) has been questioned.

Moreover Sanders type 2 fractures are two-part fractures but the situation of the fundamental fracture line is crossing the articular surface medially, laterally or in its middle. In type 2a and 2c, the fracture line respect the articular surface which remains intact; on the opposite, in type 2b, there is a trans-articular fracture with the risk of residual step off after treatment. Indeed, the 3 subtypes of type 2 fractures are very quite different.

To address these limitations, Goldzak proposed a simplified version of the Utheza classification based on a 3D volume rendering (3D VR) analysis of the fracture [7, 8]. The Utheza classification was published in the early 90’s in the French literature and remained complicated and mainly used in France.

The simplified classification proposed by Goldzak, which is based on two main types depending on the situation of the fundamental fracture line on the 3D reconstructions, aims to be easier to use and of prognostic value.

To evaluate the accuracy of Goldzak classification, we decided to compare it to Sanders classification. We hypothetized that 3D volume rendering and extra-articulation used for Goldzak classification could provide better interobserver and intraobserver reliability than with Sanders classification.

## Material and methods

### Patients

The CT scans of 30 patients with DIACFs treated in one trauma center (Department of Traumatology and Orthopedic Surgery, Ostrow Wielkopolski, Poland) between 2014 and 2018 were collected and photographed by one of us (MF). The study was approved by our institutional review board. Signed consent was obtained from all participants for all aspects of this study.

Sixteen medical doctors (5 senior orthopedic surgeons, 4 orthopedic surgery residents, 4 trauma radiology specialists, 3 radiology residents) were recruited to analyze and classify the cases using Sanders and Goldzak classification systems. One senior orthopedic surgeon and 2 trauma radiology specialists were based outside of the trauma center. Head of the department who selected the patients and CT scans did not participate in the trials.

Each observer was provided with a package consisting of 2 CT slices (one coronal view including sustentaculum tali, and one axial view including the anterior facet) for classifying according to the Sanders scoring system and 3D VR calcaneal fracture reconstruction pictures (anterior, superior, medial and lateral views) for classifying according to the Goldzak scoring system. All digital images were anonymized and no data allowed to identify both patient and treating surgeon. The images were sent on two separate sessions 8 weeks apart. To minimize the chance of observers memorizing the data from the first session, the order of the cases was randomized for the second session, and the observers were asked not to discuss their cases and classifications. Figures describing Sanders and Goldzak classifications were provided to each participant during each session.

### Sanders classification

This classification was the CT scan classification developed by Sanders et al.[9]. The grading of the intraarticular displacement of the posterior facet was based on a coronal CT scan slice and an axial cross section. The injury is classified into 4 major types depending on the number of fracture lines. Each major type is than further categorized into 3 subtypes depending on the situation of the primary fracture line.

### Goldzak classification

**Fig. 1:**
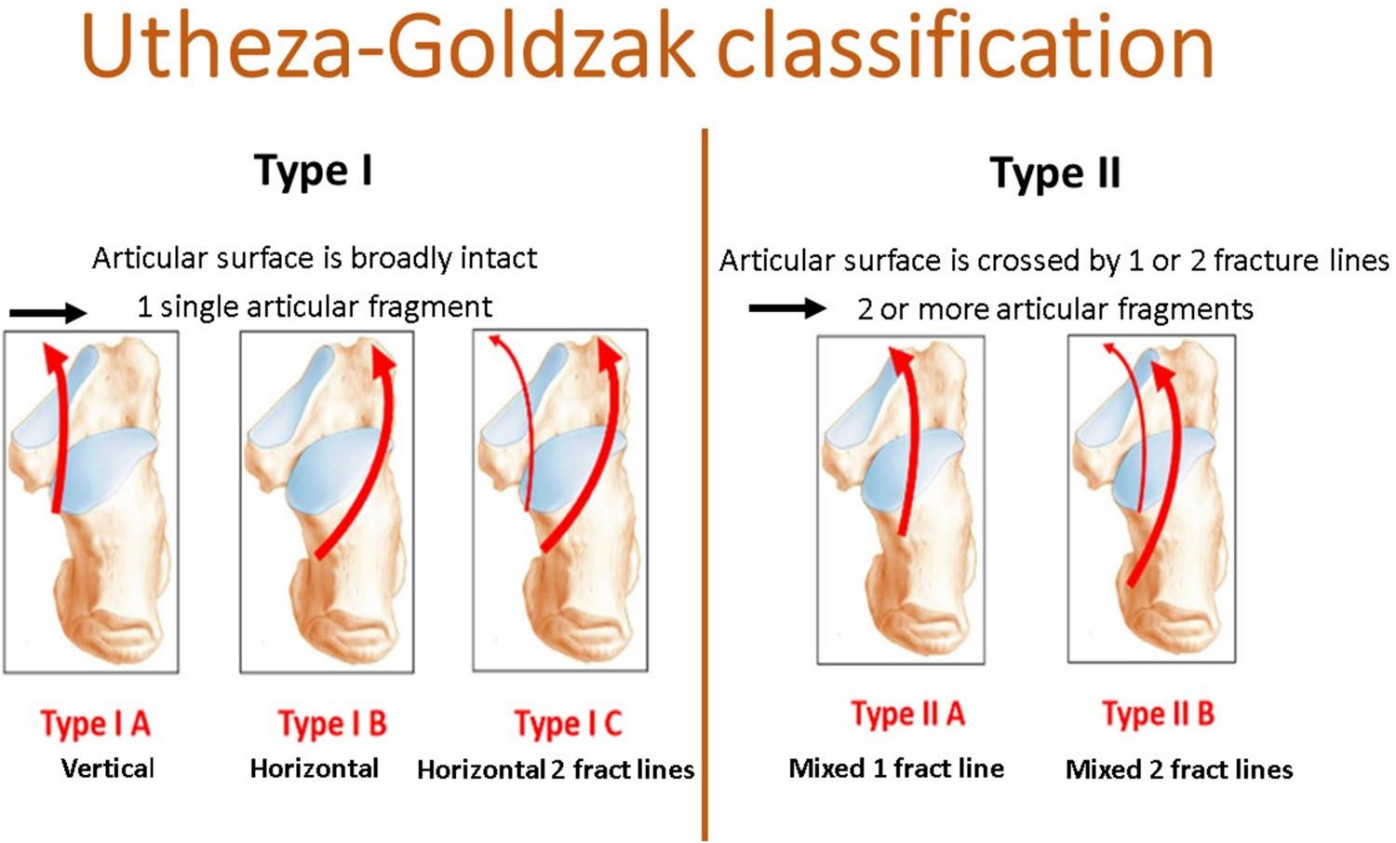
Utheza–Goldzak classification

This classification is based on the same principles as the Utheza classification—a 3D reconstruction (Fig. 1). To improve the view of the joint surface, talar and cuboid are digitally removed. Utheza then evaluated the situation of the fundamental fracture line which may be medial with a vertical displacement of the whole posterior facet, lateral with an horizontal displacement of the posterior facet, or crossing the articular surface of the posterior facet creating a double displacement. The Utheza classification also takes into account the end of the fundamental fracture line (similarly as the Essex-Lopresti [10]), describing propagated and included fractures.

**Fig. 2:**
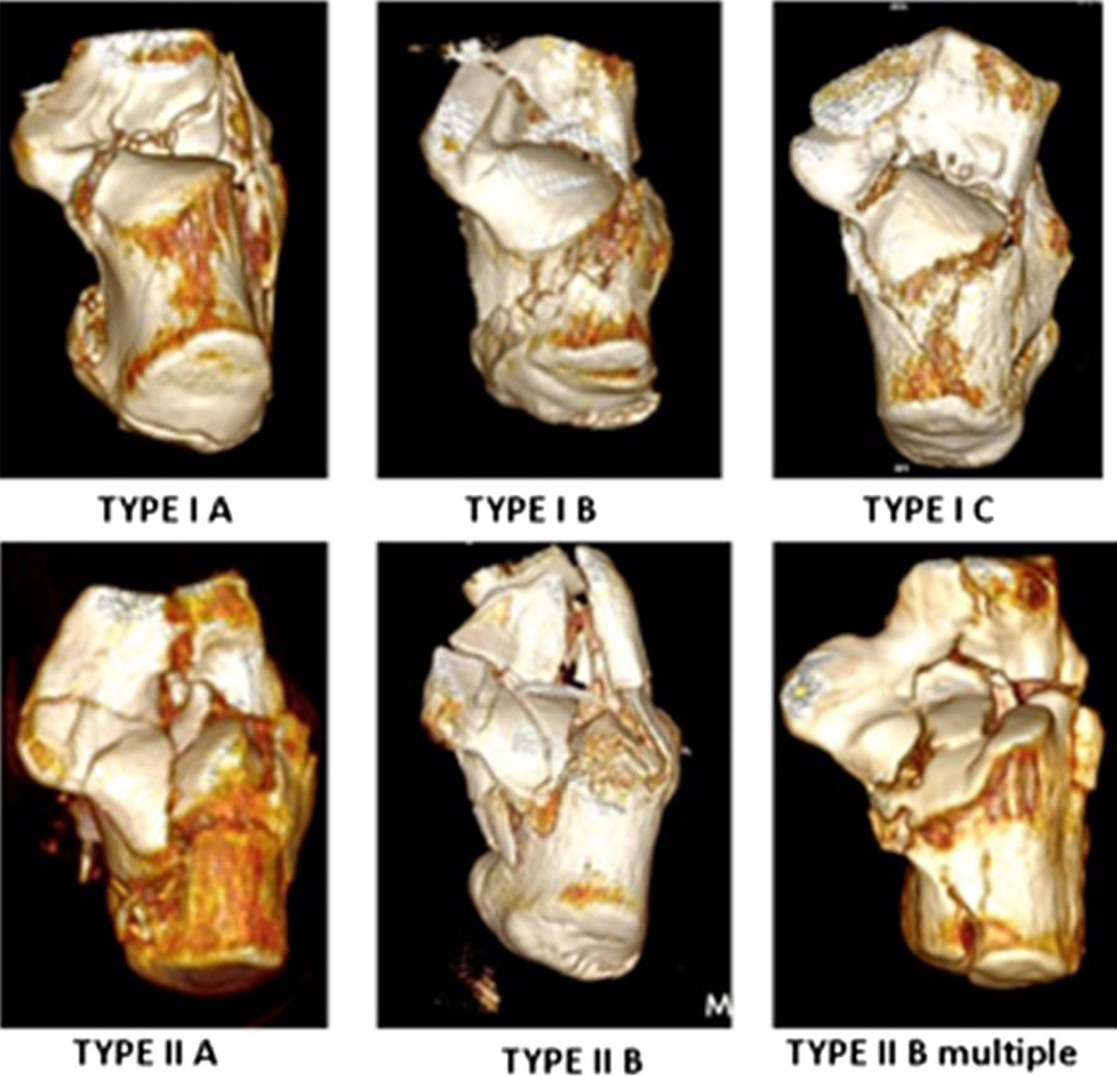
Goldzak classification

Goldzak simplified and proposed to separate two types of fractures depending on the situation of the primary fracture line, related to the posterior facet. For type 1 fractures, the posterior facet is displaced but intact, in one piece, so that the reduction should be easy and the expected result good. There are 3 subtypes depending of the situation of the primary fracture line (A medial, B lateral, C lateral with an additional fracture line between the posterior facet and the sustentaculum tali). In type 2, the fracture line crosses the posterior facet near the middle, creating a double displacement There are 2 further subtypes, 2A if there is a single fracture line which crosses the posterior facet or 2B if there are 2 fracture lines which cross the articular surface (Fig. 2).

### Statistical analysis

Intraobserver reproducibility and interobserver reliability were assessed using kappa statistics (SAS software, SAS Institute Inc, Cary, North Carolina, USA). A kappa value of 0.00 represents agreement by chance alone while a kappa value of 1.00 represents perfect agreement. Kappa statistics and Gwet’s AC1 coefficient were interpreted using the guidelines proposed by Landis and Koch [11]: 0.00 < *k* < 0.020 indicate slight agreement, 0.21 < *k* < 0.40 fair agreement, 0.41 < *k* < 0.60 moderate.

For similar studies, Furey used 30 fracture data analyzed by 4 observers while Lauder used 36 fracture data analyzed by 8 observers. As for us, we used 30 fracture data analyzed by 16 observers.

## Results

### Interrater reliability

The interrater reliability using Gwet coefficient of agreement was 0.36 (95% Cl; 0.228–0.498) for Goldzak classification and 0.30 (95% Cl; 0.200–0.393, *p* < 0.001) for Sanders classification (Table 1).

**Table 1:** Interobserver Reproducibility (Gwet’s AC1 Coefficient of Agreement)

Classification	Mean (95%CI)
Sanders	0.297 (0.200–0.393)
Goldzak	0.363 ( 0.228–0.498)

According to Landis and Koch, these results indicate fair assessment for both classifications [11].

When the subclasses were not used, a substantial assessment was found for Goldzak classification with a Gwet coefficient of 0.61, and a moderate assessment for Sanders classification with a Gwet coefficient of 0.46. When the ordinal range was taken into account, a substantial assessment was found for both classifications with, respectively, 0,61 and 0,68 for Gwet analysis.

For Goldzak classification, the Gwet coefficient was 0.29 for orthopedic residents, 0.37 for orthopedic surgeons, 0.37 for radiologists and 0.68 for radiology residents. For Sanders classification, the scores were 0.28 for orthopedic residents, 0.30 for orthopedic surgeons, 0.44 for radiologists and 0.26 for radiology residents (Table 2).

**Table 2:** Interobserver reliability (Gwet’s AC1 Coefficient of Agreement)

Classification	Sanders	Goldzak
Orthopedic surgeons	0.30	0.37
Orthopedic residents	0.28	0.29
Radiologists	0.44	0.37
Radiology residents	0.26	0.68

### Intrarater reliability

Overall, the intrarater reliability kappa coefficient was 0.60 for Goldzak classification and 0.69 for Sanders classification, which indicates that both classification systems are characterized by substantial intraobserver agreement (Table 3).

**Table 3:** Intraobserver reproducibility (Cohen’s Kappa)

Classification	Mean (95%CI)	Range
Sanders	0.698 (0.524–0.832)	0.305–1.000
Goldzak	0.610 (0.439–0.790	0.181–1.000

## Discussion

In the last five decades, 49 classification systems of calcaneal fractures have been proposed, and 19 of them are considered as current [12]. Such multitude of classifications can make the communication between radiologists and/or surgeons (or between different trauma departments) difficult.

Although the most commonly used system is the one proposed by Sanders[9], others are also frequently used worldwide [1]: Orthopaedic Trauma Association (OTA) classification [13], Essex-Lopresti [10], Crosby [14], Zwipp[15] and Regazzoni [16]. All current classification systems of calcaneal fracture are based on CT scans. However, none of these systems, including the Sanders classification, has reached the general acceptance as a result of low interrater [17]. For this reason searching a classification which would be not only descriptive but useful for surgical decisions, communication and predictive for outcome is always of value.

In this study, we compared the interobserver and intraobserver reliability of two classifications systems for intraarticular calcaneal fractures: the one of Sanders, based on 2D scans, and the Goldzak classification, based on 3D CT VR and virtual exarticulation. Both classifications considered the severity of the fracture: for Sanders, the main point is the number of fracture lines and thus the number of separated parts; for Goldzak, the main point is the preservation (or lack thereof) of the articular surface of the posterior facet. In both systems, type 1 is less severe, while type 2 for Goldzak classification and types 2, 3, 4 for Sanders classification are more severe.

In this study, interobserver rating showed fair agreement for both classifications. When the subclasses were not used (i.e., the letters were omitted), the Goldzak system showcased a substantial agreement with a 0.61 coefficient versus a moderate assessment only for Sanders classification with a 0.46 coefficient. It should be taken into consideration that the Sanders classification is well known and widely used in daily practice (especially by specialists in radiology and orthopedics), in contrast to the Goldzak classification, which was often first used by the observers during the presented study.

Among the group of radiology residents, who most likely classified less calcaneal fracture than more experienced radiology specialists, Goldzak interobserver reliability reached substantial agreement (coefficient of 0.68), while the Sanders system was characterized by only fair compatibility (coefficient of 0.26). This result suggests that the Goldzak system is more intuitive and easier to learn than Sanders. Repetitive training of observers on a classification system may further improve its reliability [1], which can result in a greater interobserver rating for Goldzak classification.

The interobserver reliability of Sanders classification in the available literature ranges from *k* = 0.22 [18] to *k* = 0.55 [5], which is classified as fair or moderate compatibility [1–3, 5, 6, 18, 19]. That observation is in line with our results, where the kappa value for those system was 0.30 (fair agreement). Brunner et al. noted, that the more complex and precisely the classification system is (i.a. Sanders), the more it may affect its reproducibility [1]. Hence, its usefulness (especially using the subtypes, A, B, and C) has come into question. Moreover, when using the Sanders system without subclasses, the interobserver agreement is greater, but still has remained stable on kappa value < 0.6 [5, 6]. Similar correlation was observed in the presented study. In the Sanders classification, the historic single CT cut, as originally described, represents the anterior portion of the posterior facet at the level of the sustentaculum tali. It is clear that it does not give a general overview at a glance of the anatomy [20] and a fracture may be identified as a Sanders type II anteriorly, as well as a Sanders type III posteriorly, depending on the chosen slice.

3D reconstruction of CT scans was proposed to improve accuracy and precision of the evaluation of fracture pathology [17]. Many authors have postulated that complex intraarticular fractures would be best classified with the use of advanced modalities 3D VR [4]. Obtained results displayed greater interobserver reliability, especially among inexperienced radiologists, when interpreting the scans using 3D CTs. Similar results were presented by Misselyn et al. Within the group of less experienced observers, they noted that the agreement with the reference standard (in Sanders system) was significantly higher when using 3D technique in comparison to 2D images [20]. Brunner et al. also found that 3D reconstruction was beneficial for less experienced doctors [1]. As there is no extra cost or radiation exposure, 3D CTs can be proposed as a valuable method with preoperative teaching and planning particularly for less experienced observers [21].

On the other hand, the interobserver reliability for both studied classification is fair. It seems that the analysis of the fractured calcaneus on 3D VR scans (i.a. Goldzak) is not always superior to 2D slices (i.a. Sanders), notably in the group of experienced radiologists, which was also highlighted in the study by Veltman et al.[18].

In the discussed study, radiology specialists gained a comparable interobserver reliability rate in the Goldzak system, and significantly higher rate in Sanders in comparison to orthopedic surgeons’ results. On the contrary, Schepers et al. observed a significantly greater coefficient value among surgeons in comparison to radiologists while classifying the calcaneal fractures according to Sanders (0.52 vs. 0.30) [12]. Thus, the modified Utheza system seems to be a more universal device for communication between radiologists and surgeons. More experienced observers achieved better single kappa values compared with less experienced observers (residents), which was reported previously [2].

The intraobserver reproducibility for Goldzak system (0.61), as well as Sanders system (0.70), was identified as substantial agreement. Similar results were presented by Lauder et al., where the Sanders system with subclasses reached moderate (0.57) agreement (substantial agreement in the 0.77 range when excluding subclasses) [5]. In the study by Veltman et al., the intraobserver rate for the Sanders classification was lower (0.46) than observed in the aforementioned results [18].

Our findings regarding 3D images are unexpected, as 3D images provide more details than CT scans and hence should make fracture classification easier. These results may be due to more common use of the Sanders classification and consequently, greater familiarity with this system. 3D CT scanning and VR with virtual exarticulation is also relatively new option, which very likely has a lot of benefits not revealed in available trials and may give useful information not captured by current classification systems [1]. Goldzak 3D VR reconstruction may enable better assessment of fragment size, displacement pattern, reliability to repair and exact fracture geometry compared with 2D; however, these issues were not the focus of this paper and require further investigation.

## Conclusions

The interobserver reliability was fair for both classification systems, while the intraobserver reproducibility was identified as substantial. Despite the better view of the fracture lines provided by 3D reconstructions, the study failed to prove the superiority of Goldzak classification compared to Sanders classification.
